# Natural selection on floral volatiles and other traits can change with snowmelt timing and summer precipitation

**DOI:** 10.1111/nph.20157

**Published:** 2024-09-27

**Authors:** John M. Powers, Heather M. Briggs, Diane R. Campbell

**Affiliations:** ^1^ Department of Ecology and Evolutionary Biology University of California Irvine CA 92697 USA; ^2^ Rocky Mountain Biological Laboratory Crested Butte CO 81224 USA; ^3^ College of Science University of Utah Salt Lake City UT 84102 USA

**Keywords:** adaptive plasticity, drought, floral scent, natural selection, pollination, seed predation, snowmelt manipulation, volatile organic compound

## Abstract

- Climate change is disrupting floral traits that mediate mutualistic and antagonistic species interactions. Plastic responses of these traits to multiple shifting conditions may be adaptive, depending on natural selection in new environments.
- We manipulated snowmelt date over three seasons (3–11 d earlier) in factorial combination with growing‐season precipitation (normal, halved, or doubled) to measure plastic responses of volatile emissions and other floral traits in *Ipomopsis aggregata*. We quantified how precipitation and early snowmelt affected selection on traits by seed predators and pollinators.
- Within years, floral emissions did not respond to precipitation treatments but shifted with snowmelt treatment depending on the year. Across 3 yr, emissions correlated with both precipitation and snowmelt date. These effects were driven by changes in soil moisture. Selection on several traits changed with earlier snowmelt or reduced precipitation, in some cases driven by predispersal seed predation. Floral trait plasticity was not generally adaptive.
- Floral volatile emissions shifted in the face of two effects of climate change, and the new environments modulated selection imposed by interacting species. The complexity of the responses underscores the need for more studies of how climate change will affect floral volatiles and other floral traits.

## Introduction

Global climate change is causing rapid changes in environmental conditions, such as increased average temperatures, more frequent extreme temperatures, and alterations of precipitation patterns (Pörtner *et al*., 2022). Those environmental changes have the potential to alter traits of organisms in ways that may influence species interactions. Average trait expression in a population can respond to the environment either directly or through evolutionary change. The former mechanism is phenotypic plasticity, in which the phenotype associated with a particular genotype responds directly to the environmental conditions (Bradshaw, 1965). In the latter mechanism of evolutionary change, the environmental change alters natural selection on the trait (Siepielski *et al*., 2009, 2017; Bemmels & Anderson, 2019), or on the ability of the trait to respond plastically, leading to an evolutionary change if trait variation is at least partly heritable (Gomulkiewicz & Shaw, 2013; Carlson *et al*., 2014).

In plants, floral traits play crucial roles in interactions with animals, and like other traits may be affected by climate change. Pollinators are thought to be the main source of natural selection on floral traits (review in Harder & Johnson, 2009), and traits can also influence interactions with natural enemies such as florivores and seed predators (Galen & Cuba, 2001; Frey, 2004; Sletvold *et al*., 2015). Some floral traits, such as floral size, show relatively consistent plastic responses to drought (review in Kuppler & Kotowska, 2021) or other environmental changes expected under climate change. In addition to trait expression, natural selection on floral morphology can change with climatic factors (Campbell & Powers, 2015). A change in selection with adverse abiotic conditions could happen in several ways. Increased resource limitations on seed production can weaken selection mediated by pollinators, as suggested for *Ipomopsis* with earlier snowmelt (Campbell & Powers, 2015). A drop in pollinator availability at a new time of flowering can strengthen pollen limitation and selection for attractive traits. Selection can shift due to changing pollinator preferences in response to plastic changes in floral traits (Dorey & Schiestl, 2022) or the availability of nectar or pollen resources.

Along with flower size, reward production, and petal color, floral scent emissions are also intimately involved in interactions with animals (Raguso, 2008). Flowers often emit a complex blend of many volatile organic compounds (hereafter volatiles; Dudareva *et al*., 2013), and a variety of insects not only detect these compounds but also show preferences or avoidance of particular volatiles or mixtures (Galen *et al*., 2011; Kessler *et al*., 2013; Byers *et al*., 2014). However, as pointed out by a recent meta‐analysis (Kuppler & Kotowska, 2021), we know less about how floral volatiles respond to environmental conditions including drought (Burkle & Runyon, 2016, 2017; Glenny *et al*., 2018; Rering *et al*., 2020; Descamps *et al*., 2021; Dötterl & Gershenzon, 2023) than we do for other floral traits. Furthermore, natural selection on floral volatiles is rarely measured, possibly due to the difficulty of doing so in the field (Parachnowitsch *et al*., 2012; Kessler *et al*., 2013; Gross *et al*., 2016; Chapurlat *et al*., 2019; Gfrerer *et al*., 2021; Campbell *et al*., 2022a). To our knowledge, there are no studies of how natural selection on floral volatiles is altered by the environmental change expected under climate change. We also know little about whether plasticity in floral volatiles is adaptive. Concordant changes in volatile emissions and fitness in a new environment would indicate adaptive plasticity (Caruso *et al*., 2006).

We focused on phenotypic plasticity of floral volatiles, and how selection on volatiles, morphology, and rewards changes in response to two features of climate change: earlier snowmelt in the spring and changes in precipitation during the growing season. Our study system was the well‐studied *Ipomopsis aggregata*, pollinated primarily by hummingbirds, but also visited by insects (Price *et al*., 2005). This species emits > 50 identifiable floral VOCs (Irwin & Dorsett, 2002; Bischoff *et al*., 2014), a few of which have been shown to influence fitness components in this species or a close relative. Emission of the floral volatiles α‐pinene and β‐pinene influences seed initiation (Campbell *et al*., 2022a), suggesting pollinator‐mediated selection on scent along with floral morphology and color (Campbell, 1989; Campbell *et al*., 1996; Meléndez‐Ackerman & Campbell, 1998). Pinene emissions also influence the proportion of fruits escaping seed predation by a fly, *Delia* (Anthomyiidae), that attacks seeds before dispersal (Campbell *et al*., 2022a), and may affect fly oviposition (Irwin & Dorsett, 2002). Pollinator‐mediated selection on the floral volatile indole emitted by *Ipomopsis tenuituba*, a close congener of *Ipomopsis aggregata*, is supported by behavioral attraction of hawkmoth visitors to that compound (Bischoff *et al*., 2015). Similar preferences of mutualists and antagonists for *Ipomopsis* floral traits could create conflicting selection pressures, so we investigated reproductive success due to both pollination and avoidance of seed predation.

In many snow‐dominated ecosystems at high elevations or latitudes, warmer spring temperatures are accelerating snowmelt, at the same time that summer droughts are changing in duration and severity (Clow, 2010; Pederson *et al*., 2011; Klein *et al*., 2016; Wadgymar *et al*., 2018). Earlier snowmelt produces a longer drought period in early summer before the onset of the summer monsoon rains (Sloat *et al*., 2015). That change in snowmelt has weakened selection on some aspects of floral morphology in *I. aggregata* (Campbell & Powers, 2015), likely due to increased water limitation preventing high seed production even when pollination is increased by a favored flower trait. Dry summer periods without precipitation are also becoming longer in the region (Zhang *et al*., 2021). A controlled drydown experiment showed that the amounts and composition of *I. aggregata* floral volatiles respond to reductions in soil moisture over 5–13 d (Campbell *et al*., 2019), but plasticity over longer time periods under natural conditions has not been characterized. Using field manipulations of snowmelt timing and summer precipitation also used by Powers *et al*. (2021) to demonstrate plasticity in floral morphology and rewards, we asked the following questions.

1. How does an advancement in timing of snowmelt and an increase or decrease in summer precipitation influence emissions of floral volatiles?
2. To what extent are effects on floral volatiles driven by changes in soil moisture?
3. Does natural selection on volatiles and other floral traits by pollinators and seed predators vary with changes to snowmelt and precipitation? If water limitation weakens selection based on seed production, we would expect weaker selection with early snowmelt and reduced precipitation.
4. Are plastic responses of traits to different environmental conditions adaptive?

## Materials and Methods

### Study system

*Ipomopsis aggregata* ssp. *aggregata (Pursh) V. E. Grant* is an herb that is widespread across montane to subalpine habitats of the western United States (Grant & Wilken, 1986). Our study site was located at Maxfield Meadow, a dry open subalpine meadow 1.0 km south of the Rocky Mountain Biological Laboratory (RMBL) in Gunnison County, Colorado, USA, at 38.9495°N, 106.9908°W and 2880 m above sea level in the West Elk Mountains. In this region, *I. aggregata* plants spend 2–10+ yr as a vegetative rosette, after which they put up a flowering stalk, flower during a single season, and die, with only rare cases of iteroparity (Campbell, 1997). Plants are self‐incompatible and require pollinators for seed production, with 94% of visits to the tubular flowers made by hummingbirds and the remainder by insects (Price *et al*., 2005).

As the plants are monocarpic, seed production in a single year provides an estimate of female fitness. Seed production relates to pollination success in this species because pollen receipt on stigmas increases with pollinator visitation rate (Engel & Irwin, 2003; Price *et al*., 2005), seeds formed increases with pollen receipt on stigmas (Campbell, 1991), and seed set is pollen‐limited (Campbell, 1991; Campbell & Halama, 1993; Campbell *et al*., 2022b). Predispersal seed predation by flies (*Delia* sp., Anthomyiidae) is common, with 10–30% of fruits attacked near our sites. Female flies deposit a single egg per flower between a sepal and petal, and the larva typically consumes all of the seeds in the developing fruit and exits as a pupa (Brody, 1997).

### Snowmelt and precipitation manipulations

To simulate two aspects of future climate change that affect water availability and its timing throughout the snow‐free growing season, we established a split‐plot experiment. Detailed methods for these manipulations and measurements of precipitation are provided in Powers *et al*. (2021).

Snowmelt timing was manipulated at the whole plot level by applying black shade cloth to three 7 m × 7 m plots to accelerate snowmelt, and leaving three other plots unmanipulated. Each whole plot was split into four 2 m × 2 m subplots, with a buffer around the subplots, that received different levels of summer precipitation (200% of normal water addition, 50% reduction with a rainout shelter, mock rainout with only the shelter structure, or unmanipulated control). Traits were measured over 3 yr, 2018–2020. Snowmelt in control plots occurred on day 119 in 2018, 158 in 2019, and 126 in 2020 (on average; Supporting Information Fig. S1). The snowmelt manipulation accelerated the date of snowmelt, making it earlier by 3–11 d (6 d on average; Fig. S1).

### Floral volatile emissions

Volatile organic compounds were sampled from single flowers using dynamic headspace sampling and analyzed with thermal desorption gas chromatography–mass spectrometry (TD‐GC‐MS) on a Shimadzu QP2020 with a Rtx‐5MS column (full methods in Campbell *et al*., 2019). A 7 cm × 10 cm bag enclosed a flower, which equilibrated for 30 min before 15 min of sampling at 100 ml min^−1^ onto 5 mg Tenax. Due to their proximity to a flower, flower buds were also included in the sample bag for 17% of samples. All sampling was done between 09:00 and 15:00 h, with 90% of samples completed before 12:00 h, the end time for daytime samples of previous studies (Bischoff *et al*., 2014). The mean sampling time was similar among treatments (10:30–10:45 h), so treatment is not conflated with temperature or solar radiation. Sampling occurred over the entire flowering period (15 dates in 2018, 8 in 2019, 6 in 2020; Fig. S2). On each day of sampling, plants with at least one open flower were sampled, from as many treatments as possible (Fig. S2), resulting in 456 samples from 374 plants (Table S1), with one to five repeated samples per plant on different flowers (16% of plants had multiple measurements). At least one ambient air control was taken per sampling date (34 samples in total). We measured soil volumetric water content (VWC) next to each plant at the time of sampling with a 12 cm soil moisture probe (HydroSense II; Campbell Scientific, Logan, UT, USA), and averaged measurements across repeated samples of each plant (Fig. S3). To calculate an average over the growing season, we also measured soil moisture at the corners and center of each subplot weekly (Fig. S3), which differed across the snowmelt and precipitation treatments (Powers *et al*., 2021).

Volatile compound identification, filtering, and quantitation generally followed Campbell *et al*. (2019). Filtering was performed in the bouquet R package (Eisen *et al*., 2022). To be included in further analyses, a compound must have a retention time between 2 and 17 min, be detected in 10% of samples, and the mean in floral samples must exceed four times the mean in ambient controls. After manually removing 20 suspected contaminants, quantitative integrations were established for the remaining 29 volatiles, including set retention time windows, target ions to integrate, and two required reference ions. Retention indices were compared with published values. Quantitation employed dilution series of seven standards (9–28 replicates per dosage) run in each year of GC‐MS analysis: (*1R*)‐(+)‐α‐pinene, β‐caryophyllene, (*E*,*E*)‐farnesol, (Z)‐3‐hexen‐1‐ol, indole, linalool, and methyl salicylate, with each compound quantified using the standard of the same compound class. This yielded a filtered dataset of emission rates per flower per hour for each floral sample; we did not analyze relative amounts. Emission rates were averaged across repeated samples of each plant before analysis.

### Floral morphology and rewards

Measurements of other floral traits included corolla length, corolla width at the opening, style length, sepal width (in 2019 and 2020 only), nectar production, nectar concentration, and maximum inflorescence height. Multiple measurements of a plant were averaged before analysis. Depending on the trait, 337–443 flowering plants were sampled across the 3 yr and all treatments (Table S1), with detailed techniques reported in Powers *et al*. (2021). While measurements were attempted for all flowering plants in the experiment, only 228 plants (6–28 plants per treatment per year) had complete information on all these traits and floral volatiles, due to the irregular availability of open flowers or buds on each sampling day.

### Fitness measures

We assessed female fitness on each flowering plant using four measures. First, overall female fitness was assessed as total seeds produced by the plant. This metric is straightforward but closely correlated with flower number, a resource‐related trait that is determined before interactions with pollinators or seed predators occur. Second, we estimated a fitness component that captures pollination success without the effect of seed predation: the number of seeds initiated per flower (Campbell *et al*., 2022a). Third, to quantify the fitness effect of seed predators, we examined the proportion of fruits attacked by flies, or more rarely by caterpillars (Noctuidae; Juenger & Bergelson, 1998). To turn the latter into a fitness component representing escape from seed predation, we calculated 1 minus the proportion attacked (Campbell *et al*., 2022a). Finally, we measured the lack of resistance to fly oviposition by determining the average number of fly eggs per flower.

Fruit and seed production, along with seed predation, were assessed by collecting fruits just before dehiscence and the calyces from flowers that failed to make a fruit (i.e., aborted), following methods used in previous studies with this species (Campbell, 1991; Campbell *et al*., 2022a), except that sollections were made less often to minimize environmental impact (every 6–8 d) over the entire fruiting period (dates in Fig. S1). Seed production in undehisced fruits was counted directly. Seed production by fruits that had already dehisced and spilled their seeds was estimated as the average seeds per intact fruit for that plant (Campbell *et al*., 2022a). The few flowers collected early for measurement were given values for seed production equal to the average for flowers that had been allowed to set seed on the plant. Fruits filled with frass indicating seed predation by a fly larva generally had zero viable seeds. Fly and caterpillar damage was calculated as the proportion of nonaborted fruits with fly frass (14% of fruits) or eaten by a caterpillar (2% of fruits). The number of flowers per plant was the total of damaged and intact fruits, aborted flowers, and flowers collected early or for trait measurement. Seeds initiated per flower were calculated for each plant as the product of nonaborted fruits per flower (whether the fruit was eaten or not) and seeds per fruit for noneaten fruits.

To estimate the incidence of fly oviposition, we surveyed all tagged flowering plants across the entire flowering season (dates in Fig. S1). Each week, we counted the number of fly eggs laid under the sepals on all open flowers and elongated buds. We then summed these counts across the season and divided the total eggs by the total number of open flowers and buds to yield fly eggs per flower.

### Statistical analyses

#### Questions 1 and 2: Impacts of snowmelt, precipitation, and soil moisture

We analyzed responses of floral volatile emissions to snowmelt timing and summer precipitation (Q1) in two ways. First, we modeled the response to the experimental manipulations and years coded as discrete levels of the replicated split‐plot design (Table 1A). Second, we ran a multiple regression against two continuous environmental variables, the date of snowmelt in each plot and summer precipitation estimated for each precipitation treatment (Table 1B). These variables are affected by both the treatments and natural variation among years. The first model tests for causal effects of the treatments, and the second model expands the range of snowmelt timing and summer precipitation to increase statistical power, albeit in a correlational analysis. Detailed rationales and comparison of these two modeling approaches are given in Powers *et al*. (2021). To determine the extent to which impacts on floral traits are mediated by soil moisture (Q2), a third model related emissions to year and the soil moisture. Because the timescale of how volatiles respond to water is unknown, we used two measures of soil moisture that differed in scale: near the plant on the day(s) of sampling (Table 1C), or averaged across the flowering season and 2 m × 2 m subplot (Table 1D).

**Table 1:** Analyses of plasticity of *Ipomopsis aggregata* floral volatiles emissions.

	df	*F*	*P*
(A) Treatments			
**Year**	**2**	**11.7**	**0.001**
Precip	2	1.3	0.145
Snow	1	1.1	0.287
Year × Precip	4	1.0	0.494
**Year × Snow**	**2**	**1.6**	**0.048**
Precip × Snow	2	1.1	0.358
Year × Precip × Snow	4	0.8	0.761
13% explained			
(B) Environmental variables			
**Snowmelt date**	**1**	**9.2**	**0.001**
**Summer precipitation**	**1**	**2.8**	**0.008**
Snowmelt × Precipitation	1	1.1	0.270
5% explained			
(C) Soil moisture measured at the plant level			
**Year**	**2**	**10.9**	**0.001**
**Soil moisture**	**1**	**2.8**	**0.008**
**Year : Soil moisture**	**2**	**1.7**	**0.028**
10% explained			
(D) Soil moisture averaged across the season at the subplot level			
**Year**	**2**	**11.7**	**0.001**
Soil moisture	1	1.0	0.455
**Year : Soil moisture**	**2**	**1.6**	**0.038**
9% explained			

(A) Responses to snow and precipitation (precip) treatments and year. (B) Responses to environmental variables of snowmelt date and estimated summer precipitation. (C) Responses to soil moisture at the plant level. (D) Responses to soil moisture at the subplot level. A canonical analysis of principal coordinates was performed for each set of explanatory variables and their interactions using a matrix of Bray–Curtis distances among volatile emission rates (*n* = 346 plants for section C, otherwise *n* = 374). Bold text indicates significant effects. The percent of the total inertia explained by the variables is given.

To assess the plastic response of volatile emission rates in each of the three models, we employed canonical analysis of principal coordinates (CAP, Anderson & Willis, 2003) with Bray–Curtis distances, as implemented in the ‘capscale’ function of the R package vegan (Oksanen *et al*., 2024; R Core Team, 2024). This constrained ordination method is suited to discover multivariate patterns among predefined predictors. We used a permutation test (‘anova.cca’ function) to test each term of the full model sequentially and test for all interactions after accounting for the main effects. As currently implemented, CAP does not allow inclusion of random effects. So for each of the three models, we supplemented the multivariate analysis with a linear mixed model of total emission rates (or the total emissions of four compound classes: monoterpenes, sesquiterpenes, benzenoids, and aliphatics) that included random effects of plot and subplot, matching the split‐plot design. Emission rates were square‐root transformed before plasticity analyses.

#### Question 3: Selection

Measuring natural selection on large numbers of traits such as volatile emissions is statistically challenging due to high dimensionality and correlations among traits that lead to problems with multicollinearity during multiple regression (Mitchell‐Olds & Shaw, 1987). Studies of selection on multidimensional phenotypes have avoided this problem by modeling selection on each trait separately, dropping traits from the analysis, using regularized regression, or reducing dimensionality with principal components analysis (Chong *et al.*, 2018) or reduced‐rank regression (examples for floral volatiles include Schiestl *et al*., 2011; Parachnowitsch *et al*., 2012; Opedal *et al*., 2022). As no one approach was ideal for our data set, we adopted three approaches to understand how selection on traits, including volatile emission rates, shifts with the environment. All selection analyses were conducted for each of the four fitness measures (relative to mean fitness across all years), with traits mean‐centered and scaled by the SD.

First, we aggregated volatiles by compound class and modeled relative fitness against (1) emissions of the four compound classes, (2) year and the snowmelt and precipitation treatments, and (3) all interactions between variable sets (1) and (2), which indicate shifts in selection among treatments or years. This method represents the experimental design well, and takes into account correlations among compound classes, but may mask selection on individual compounds.

Second, we assessed the dependence of selection on two continuous environmental variables (snowmelt date in each plot and precipitation in each subplot), mirroring the approach for the plasticity analyses. To avoid a loss of power, we characterized univariate selection for each floral trait, including 12 volatiles that occurred in 75% of samples. We modeled how relative fitness was affected by the trait, the environmental variables, and the interactions of the trait and each environmental variable, which represent shifts in selection with the environment. If both interactions were nonsignificant (*P* > 0.05), we dropped them from the model to estimate the main effect of the trait on fitness. Using the environmental variables increases power but is correlational (see Powers *et al*., 2021) and the measure of univariate selection does not separate direct selection on a trait from indirect selection from correlated traits (Lande & Arnold, 1983), which are common for integrated morphological traits and volatiles within the same biosynthetic pathway.

Third, to address this last concern we performed elastic net regression to calculate direct selection gradients on each trait. This method, a type of regularized regression, performs variable selection (selection gradients on some traits are estimated as zero, reducing model complexity) and constrains the magnitude of selection estimates in order to decrease the variance of the estimated gradients, at the cost of introducing some bias. Regularized regression has been shown to more accurately estimate the direction and magnitude of total multivariate selection when some traits are correlated (Morrissey, 2014; Gfrerer *et al*., 2021; Sztepanacz & Houle, 2024). Besides traits, we add terms for the two continuous environmental variables to capture the direct effects of the environment on fitness. Because correlations between volatile emissions and other floral traits were low (mean absolute *r* = 0.07 compared with 0.32 among volatiles and 0.27 among other traits; Fig. S4), making indirect selection between those groups unlikely, we first analyzed the two groups of traits separately. We modeled selection on the other six floral traits in a separate analysis (excluding sepal width as that trait was not measured in 2018, and flower number as that correlates strongly with total seeds and represents a fitness component rather than a trait under selection). We then performed a combined analysis with all traits, applying the elastic net approach to the subset of 241 plants for which all six traits and volatile emissions were available. Because this subset of plants is likely nonrandom (larger plants that had open flowers available on more sampling days), we do not emphasize the results of this latter approach. Elastic net regressions with ɑ set to 0.5 were run using the glmnet R package after selecting an optimum value of λ via cross‐validation (Friedman *et al*., 2010). To compare the direction and magnitude of selection in each treatment, we also ran models for each treatment subset without the continuous environmental variables (Fig. S5).

#### Question 4: Adaptive plasticity

For plasticity to be adaptive, a phenotypic change in response to an environmental change must be in the direction favored by selection in the new environment (see Ghalambor *et al*., 2007). In other cases, the plastic change can be neutral or maladaptive (decrease fitness). We constructed plots to visualize how the plasticity of a trait to precipitation or snowmelt timing related to selection in the modified environments. For example, the selection gradient of each trait under early snowmelt was graphed against the change in emissions between early vs control snowmelt (calculated as the ratio of estimated marginal means in a model that also included precipitation treatment). Univariate selection on each trait was calculated for each combination of snowmelt or precipitation treatment and year. We also checked whether the direction of direct selection matched when estimated by the elastic net method (in each subset of treatment and year, without the continuous environmental variables). We excluded fly oviposition as that is a less direct fitness measure compared with the proportion of fruits escaping seed predation.

## Results

### Q1: Impacts of snowmelt timing and summer precipitation on volatiles

The most dominant compound emitted by these flowers of *I. aggregata* was α‐pinene (28% on average), followed by minor amounts (5–12%) of limonene, (*Z*)‐β‐ocimene, 3‐carene, (*Z*)‐hex‐3‐en‐1‐ol, (*E*)‐β‐ocimene, and 23 other volatiles (< 5%; Table S2), similar to the composition in previous studies of this species (Bischoff *et al*., 2014; Campbell *et al*., 2019; Wu *et al*., 2023). Emissions of 30 compound pairs were strongly correlated (*r* > 0.50; Fig. S4). The CAP plasticity analysis of the effects of the year and the treatments on volatile emissions explained 13% of the total variation (Table 1A). Volatile emissions did not respond on average to the snowmelt or precipitation treatments (*P* > 0.14), but there was a significant interaction between snowmelt treatment and year (*P* = 0.048). The significant variation in emissions among years (*P* = 0.001) can be attributed to high α‐pinene and low 3‐carene emissions in 2019 (CAP ordination not shown). Emissions of all four compound classes differed by year (*P* < 0.02), and early snowmelt lowered aliphatic emissions (*P* = 0.044; Table S3a; Fig. S6). Across the 12 volatiles that occurred in 75% of samples, plasticity to early snowmelt was significantly correlated with plasticity to precipitation reduction in 2020 (*r* = 0.73; *P* = 0.0004; Fig. S7) but not in the other 2 yr (*P* > 0.28).

The plasticity of floral volatiles to the environmental manipulations was generally similar to the plasticity of the other traits (floral morphology, nectar traits, and inflorescence height), with mean absolute Cohen's *d* effect sizes of 0.13 for volatiles vs 0.12 for other traits with early snowmelt (Wald test *P* = 0.77), 0.19 vs 0.20 for precipitation reduction (*P* = 0.77), but effects of precipitation addition on volatiles were smaller than effects on other traits (0.12 vs 0.27, *P* < 0.001).

**Fig. 1:**
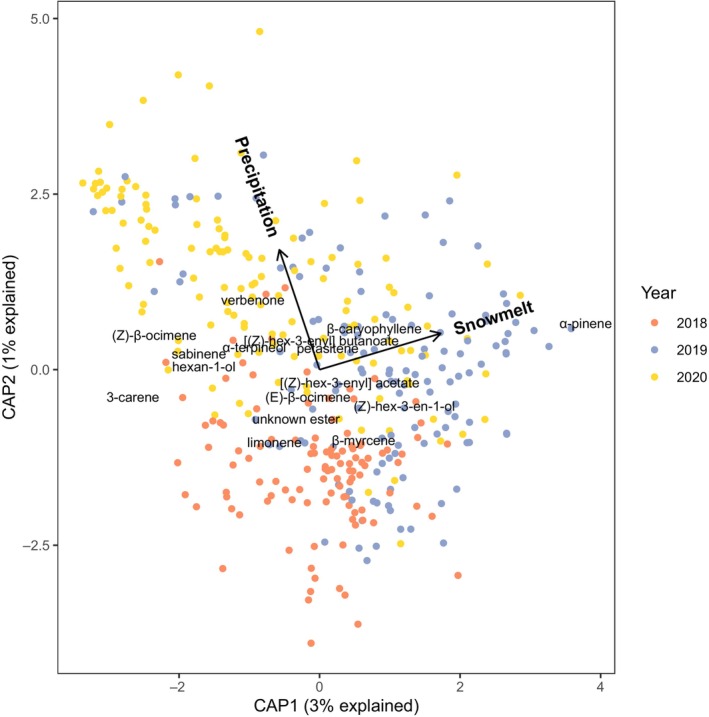
Constrained ordination of *Ipomopsis aggregata* floral volatile emissions derived from a canonical analysis of principal coordinates with snowmelt date and estimated precipitation in each subplot as explanatory variables. Points indicate individual plants. Arrows indicate the direction that each environmental variable increases, with the nonsignificant interaction dropped for visualization. Each volatile compound is labeled in the direction it increases. Only snowmelt date was significantly correlated with emission rates (Table 1B).

The CAP analysis that tested for associations between emissions and the snowmelt date and summer precipitation in each subplot explained 5% of the total variation and attributed most of that variation to snowmelt date (Table 1B). Differences among years with different snowmelt dates were driven the most by higher α‐pinene and lower 3‐carene emissions in the late snowmelt year of 2019 (Fig. 1). Higher summer precipitation was associated most strongly with higher verbenone emissions and lower β‐myrcene emissions. There was no interactive effect of the two environmental variables on volatiles (*P* = 0.27). Emissions of each compound class did not respond significantly to these two environmental variables (Fig. S8; Table S3b).

### Q2: Impacts of soil moisture on volatiles

**Fig. 2:**
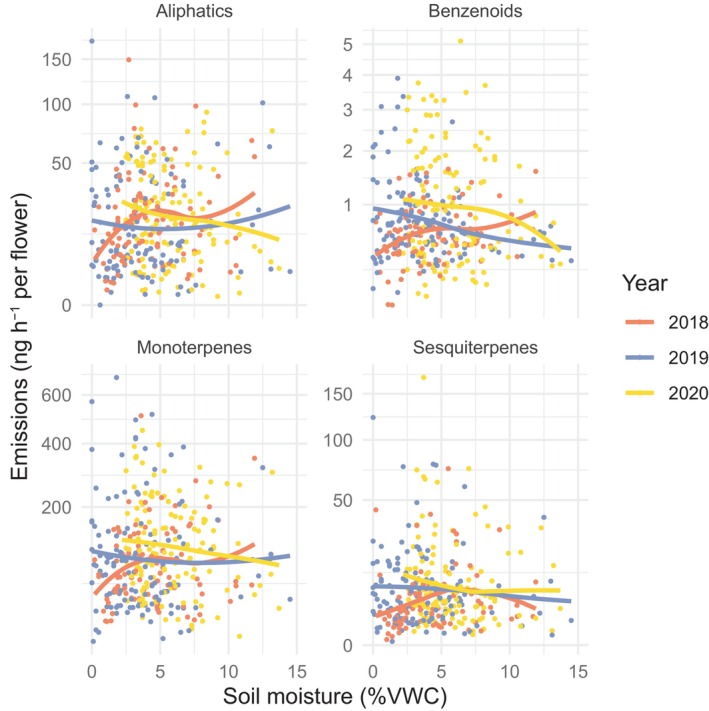
Effects of soil moisture (volumetric water content, VWC) on *Ipomopsis aggregata* floral emissions of each compound class. Soil moisture was measured next to each plant during volatile sampling. Curves show loess fits in each year.

Volatiles were compared with soil moisture at two temporal scales. At the short timescale, volatile emissions were correlated with soil moisture measured at the plant level on the day(s) of sampling (*P* = 0.008; Table 1C) although there was no average effect on emissions of each compound class (Table S3c; Fig. 2). There was a significant interaction of plant‐level soil moisture and year for all volatile emissions (*P* = 0.028; Table 1C). Grouping by compound class showed that interaction was driven primarily by changes in total benzenoids and total aliphatics (Table S3c).

At the longer timescale, volatile emissions were not correlated overall with subplot‐level soil moisture averaged through the season (*P* = 0.46; Table 1D) nor was there an effect on emissions of each compound class overall (Table S3d; Fig. S9). However, the effect of subplot‐level soil moisture on volatile emissions depended on the year (Table 1D).

### Q3: Selection on volatiles and other traits in different environments

**Fig. 3:**
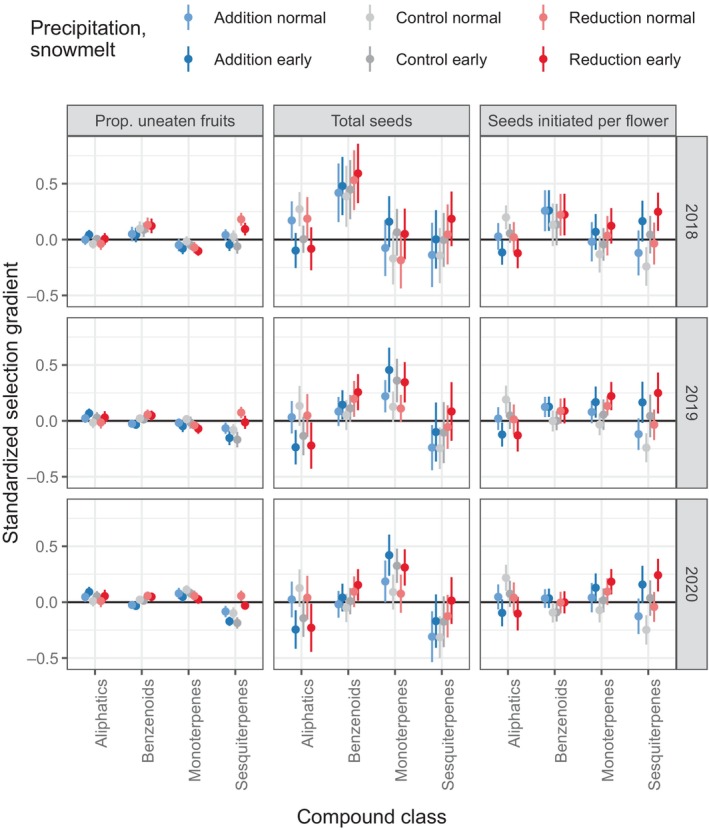
Selection on *Ipomopsis aggregata* floral volatile emission rates in each year and precipitation and snowmelt treatment. Points indicate standardized selection gradients (mean ± SE) estimated from fitness measures (columns) in each year (rows). Model results are reported in Supporting Information Table S3. No significant selection was detected for fly oviposition so that measure is not plotted.

Selection on floral traits, including volatile emission rates, was assessed in different environments with four measures of female fitness. The first analysis assessed how selection on total emissions of each class of compounds depended on the treatments and year (Table S4; Fig. 3). For fly oviposition, there was no overall selection detected on any compound class (all *P* > 0.17). For the proportion of fruits escaping seed predation, selection on monoterpenes varied by year, and selection on sesquiterpenes depended on precipitation (halved precipitation led to more positive selection). For seeds initiated per flower, there was no overall selection on any compound class (all *P* > 0.19), but selection on sesquiterpenes depended on snowmelt (early snowmelt led to more positive selection). For total seeds, selection on benzenoids was positive overall and selection on aliphatics depended on snowmelt, with more negative selection under early snowmelt. Whereas this method cleanly demonstrated some changes in selection on sesquiterpene and aliphatic emissions with the environment, it does not reveal which individual volatiles were under selection.

**Fig. 4:**
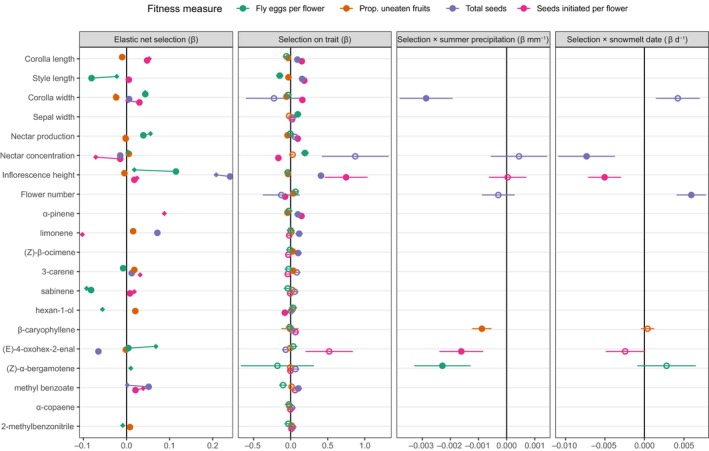
Model results for the effect of estimated summer precipitation and snowmelt date on selection gradients for *Ipomopsis aggregata* floral traits, including volatile emissions. The left panel shows direct selection on the trait estimated by the elastic net method applied separately to volatile emission rates and other traits (circles) or in a combined analysis of all traits (diamonds). Missing points indicate the trait was not selected by the model. Standard errors and *P* values are not easily available for elastic net regression (Sztepanacz & Houle, 2024). The next three panels show univariate selection (direct and indirect) on each trait (with SE) and how that selection changes with the environment: the effect of summer precipitation on selection, and the effect of snowmelt date on selection. In these panels, terms that were not significant (*P* > 0.05 in the linear model) are shown with open circles, and both interactions were dropped from the model if neither was significant. After the other floral traits, volatiles are sorted in descending order by mean emissions rate. Note that unlike the other three fitness measures, increased fly eggs per flower indicates potentially reduced fitness, as a fly larva generally consumes all seeds in the fruit.

The second selection analysis assessed the dependence of univariate selection on two environmental variables: snowmelt date and summer precipitation in each subplot (Fig. 4, right three panels). The third selection analysis accounted for trait correlations to quantify direct selection on either volatiles or other floral traits, regardless of the environment (Fig. 4, left panel). Direct selection gradients often matched the direction of those in the univariate analysis, and we report them here when their magnitude exceeds 0.05 (in either the combined or separate analyses).

For fly oviposition, we detected direct selection for hexan‐1‐ol, sabinene, and 3‐carene (flies laid fewer eggs per flower on plants with higher emissions). Direct selection favored lower emissions of (*E*)‐4‐oxohex‐2‐enal, (*Z*)‐α‐bergamotene, β‐caryophyllene, and (*Z*)‐β‐ocimene. Univariate and direct selection favored plants with long styles. Univariate selection favored narrow sepals and dilute nectar, while direct selection favored plants with short inflorescences, narrow corollas, and low nectar production. As precipitation decreased, univariate selection favored plants with lower emissions of the sesquiterpene (*Z*)‐α‐bergamotene.

For escape from seed predation, univariate selection favored plants with low α‐pinene emissions, high (*Z*)‐β‐ocimene and 3‐carene emissions, short and narrow corollas, short styles, low nectar production, and short inflorescences with more flowers. Reduced precipitation led to univariate selection against β‐caryophyllene.

For total seeds, there was univariate selection for plants with high α‐pinene, methyl benzoate, limonene, and (*Z*)‐β‐ocimene emissions, with direct selection for limonene. Univariate selection favored long corollas and styles and tall inflorescences. Direct selection also favored tall inflorescences. With less summer precipitation, univariate selection on corolla width became less negative. With earlier snowmelt, univariate selection on nectar concentration became more positive and selection for higher flower number weakened.

For seeds initiated per flower, univariate selection favored plants with high α‐pinene (β = 0.15 ± 0.04, *P* = 0.0001), and low hexan‐1‐ol emissions, and direct selection favored long corollas, dilute nectar, high α‐pinene, and low limonene. Reduced precipitation led to more negative univariate selection on (*E*)‐4‐oxohex‐2‐enal. There was again positive univariate selection for inflorescence height, and this selection strengthened with earlier snowmelt.

Magnitudes of univariate selection on the traits ranged from β = −0.23 to 0.87 and varied up to 0.003 units mm^−1^ precipitation or up to 0.007 units d^−1^ shift in snowmelt timing (Fig. 4). There were direct effects of environmental conditions on fitness (with the trait removed from this model for ease of interpretation): for each day the snow melted earlier, fly eggs per flower increased 2.2 ± 0.3%, escape from seed predation increased 0.3 ± 0.1%, total seeds declined 1.4 ± 0.2%, and seeds initiated per flower declined 1.6 ± 0.2% (all *P* < 0.001), with no detected effect of summer precipitation (all *P* > 0.05).

### Q4: Adaptive plasticity

**Fig. 5:**
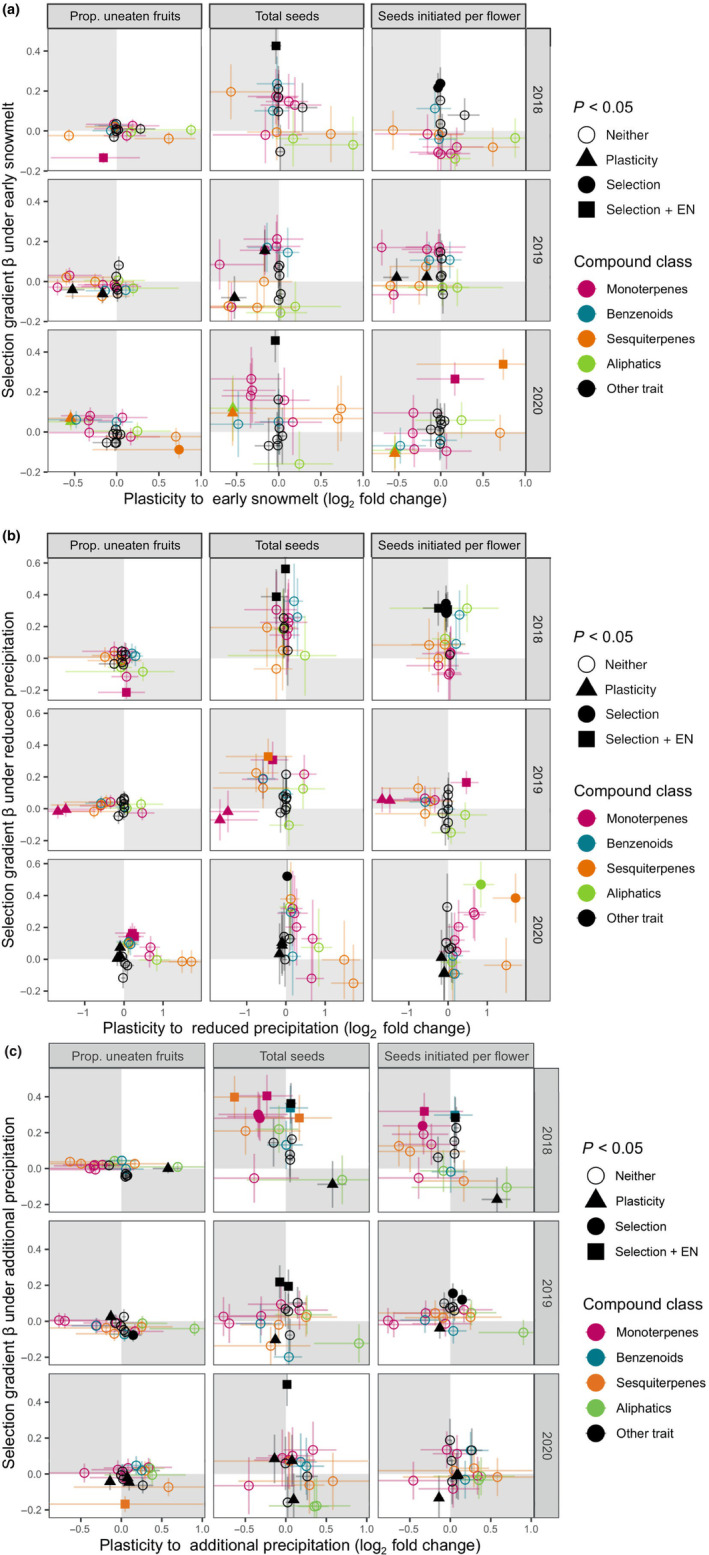
Correspondence between plasticity and selection for each *Ipomopsis aggregata* floral trait, including volatile emissions, in each of three new environments: (a) early snowmelt, (b) reduced precipitation, and (c) additional precipitation. Standardized univariate selection gradients (β) for each trait in the new environment are plotted against the plasticity calculated as the fold change between the new and old environments (unmanipulated snowmelt timing or precipitation). The volatile compound class is indicated by color. Adaptive plasticity occurs when traits are in the first or third quadrants (white), maladaptive plasticity occurs in the second or fourth quadrants (gray), and neutral plasticity occurs along the *x*‐axis. Panels correspond to the three fitness measures (proportion of uneaten fruits, total seeds, and seeds initiated per flower) and 3 yr. Error bars represent SE and shape indicates statistical significance of plasticity and selection, with squares indicating that univariate selection was significantly different from zero (*P* < 0.05 in a linear model) and the elastic net (EN) estimate of direct selection on the trait was not zero.

The direction of plasticity to earlier snowmelt or altered precipitation was concordant with the direction of selection in the altered environment, suggesting adaptive plasticity, in about half of all cases (points for each trait fall in white quadrants in Fig. 5). Across 3 yr, the number of volatiles that trended toward adaptive plasticity was roughly equal to those that showed maladaptive plasticity to each new environment (38–57% of responses adaptive depending on the fitness measure, *P* > 0.08 in a test of equal proportions). The above proportions are based simply on the sign of plasticity and selection. A more stringent test of adaptive plasticity would require that both the plasticity and the selection in the new environment be statistically significant. Univariate selection in the new environment was statistically significant 9% of the time and plasticity 8% of the time, but no floral trait showed both significant selection and plasticity in any year or treatment (Fig. 5).

## Discussion

The impacts of climate change on floral volatiles are only beginning to be explored. A few previous studies have tested for impacts of drought (Burkle & Runyon, 2016; Glenny *et al*., 2018), nutrient availability (Friberg *et al*., 2017), or temperature (Farré‐Armengol *et al*., 2014; Cna'ani *et al*., 2015), on floral volatile emissions (reviewed in Majetic *et al*., 2009; Farré‐Armengol *et al*., 2013; Dötterl & Gershenzon, 2023). Our study examined the impact of multiple aspects of climate change (snowmelt timing and precipitation) on floral volatiles and also asked how climate change influences natural selection mediated by both mutualists and antagonists in the new environment.

### Plasticity of floral volatiles in response to two climate perturbations

In a subalpine plant, *I. aggregata*, floral volatiles responded to experimental manipulations that advanced spring snowmelt by 3–11 d, with the effect depending on the year. Considering a wider environmental gradient that included interannual variation as well as treatment effects, volatiles responded to both snowmelt timing and precipitation. These changes in volatile emissions occurred in tandem with the effects of drought on other plant traits under these same manipulations. For example, lower precipitation decreased corolla length and width, style length, sepal width, and nectar production, while increasing nectar concentration (Powers *et al*., 2021). Drought also reduced stomatal conductance and increased water‐use efficiency in vegetative plants (Navarro *et al*., 2022). The plasticity of volatiles could be stronger than that of morphological traits if individual biochemical pathways are more quickly adjusted than flower development. Alternatively, resources could limit costly biomass investments but not inexpensive (in terms of carbon) floral volatile production, as in the case of nitrogen manipulations in *Lithophragma bolanderi* that appeared to have stronger effects on leaf size and flower number than on floral volatiles (Friberg *et al*., 2017). In *I. aggregata*, plasticity of volatiles to the environmental manipulations was similar in magnitude to responses in visual display and rewards.

Although precipitation and snowmelt timing both affected water availability, the responses of volatiles to early melt and summer drought only aligned in one of the 3 yr of the study. A potential reason is that the two climate variables affect soil moisture during different plant developmental stages – snowmelt during the early‐season acquisition of resources and summer precipitation while flowers are in bud or open. Snow also insulates plants from extreme spring temperatures that could affect later volatile production differently than drought. Evidence for interactive effects of two biotic or abiotic factors on floral volatiles is mixed: No interaction of drought and herbivory on total volatiles was observed across four forb species (Burkle & Runyon, 2016), but there was an interactive effect of drought and elevated CO_2_ on total volatiles for one of those species (Glenny *et al*., 2018). No dependence of precipitation effects on snowmelt timing was observed for *I. aggregata* volatiles, in contrast to such an interactive effect on floral morphology (Powers *et al*., 2021).

### Diverse responses of volatile emissions to soil moisture

Water availability varies at small temporal and spatial scales in response to snowmelt, precipitation events, infiltration, evaporation, and microsite variation. At the small scale, volatile emissions responded to plant‐level daily fluctuations in soil moisture (0–15% VWC), and these effects depended on the year. On a coarser temporal scale, drier soils averaged across the season (2–8% VWC) were not correlated with emissions. In contrast to the trend for increased total floral volatile emissions under drought in some other species (Burkle & Runyon, 2016; Glenny *et al*., 2018) and in a previous study of *I. aggregata* (Campbell *et al*., 2019) that is hypothesized to occur as a response to plant stress (Farré‐Armengol *et al*., 2013), emissions of the four compound classes did not increase with declining soil moisture. But since responses of volatiles to soil moisture can be nonlinear, it is critical to examine the responses over specified ranges of soil moisture or specified levels of drought stress (Yuan *et al*., 2009; Peñuelas & Staudt, 2010). In a controlled experiment in which potted plants of *I. aggregata* were subject to a dry down over 2 wk with no water, soil moisture fell below *c*. 5% VWC between the last two time points (Campbell *et al*., 2019). Our results in the field are similar to those in the controlled experiment for α‐pinene, the dominant monoterpene, since it fell in emission below *c*. 5% VWC in the dry down study and in the current field study in 2019 (Fig. 2). This result aligns with the hypotheses that drought could lower emissions through reduced stomatal conductance (Farré‐Armengol *et al*., 2013; Niinemets *et al*., 2013), which we also saw as a drought response in this experiment (Navarro *et al*., 2022), or decrease emissions through reduced enzyme or substrate availability (Peñuelas & Staudt, 2010). Some compounds responded differently in the field and glasshouse studies, perhaps due to environmental differences: in the field (*E*)‐β‐ocimene did not respond overall to lower soil moisture even though it increased as the dry down progressed in Campbell *et al*. (2019).

In the current study, the responses of volatiles to changes in the environment were complex and often nonsignificant despite one of the largest sample sizes (374 plants) for any floral volatile study, partly because of the high variation in emissions (Fig. 2). The environmental models explained only 5–13% of the total variation in emissions, so other sources of variation are worth investigating, including variation due to temperature (as demonstrated in this species; Wu *et al*., 2023), flower age, genetics, and resources accumulated over the plants' long lifespans. The complexity of these responses underscores the need for more field studies of plasticity and selection on floral volatiles to make general predictions about the impacts of climate change.

### Environment modulates strength of selection on volatiles and other traits

Natural selection on floral volatiles has only rarely been measured under natural conditions (Parachnowitsch *et al*., 2012; Kessler *et al*., 2013; Gross *et al*., 2016; Chapurlat *et al*., 2019; Gfrerer *et al*., 2021; Campbell *et al*., 2022a). For other plant traits, there is evidence that selection responds to environmental conditions: In a global meta‐analysis, precipitation explained a portion of the variation in selection on plants (Siepielski *et al*., 2017). In a previous *Ipomopsis* study, selection for longer corollas was stronger in years of later snowmelt, likely because greater water availability allowed the higher pollen receipt due to greater pollinator visitation to translate into mature seeds (Campbell & Powers, 2015). That hypothesis would predict stronger selection (based on total seeds) on traits in general with later snowmelt and greater summer precipitation, but we did not detect that result in this study, either specifically for corolla length or for traits in general (Figs 4, S5). For example, selection on aliphatic volatiles became negative with early snowmelt rather than merely less positive (Fig. 3). Instead, summer drought led to stronger selection for wider corollas (which were favored overall in previous studies; Campbell *et al*., 2022a, 2023) and earlier snowmelt led to stronger selection for concentrated nectar and sesquiterpene emissions (Figs 3, 4). Additionally, through unknown mechanisms, seed predator selection on sesquiterpenes was affected by early snowmelt (more negative selection; Fig. 3). One other study examined impacts of environmental conditions on selection of floral volatiles (Dorey & Schiestl, 2022), but in a growth chamber rather than the field. In that study, plants of *Brassica rapa* showed plasticity of volatiles in response to herbivory and soil type, but no selection was detected on the floral volatiles. Our study reveals the possibility of natural selection and evolution of floral volatiles changing with climate change, especially since emissions of some volatiles show evidence of genetic variance in *Ipomopsis* (Campbell *et al*., 2022c). One limitation is that our manipulations were on a small‐scale plot level, not allowing for changes in pollinator preferences or pollinator abundance that might happen with large‐scale climate change. Those mechanisms could be elucidated through a long‐term study that compared selection to annual climatic conditions (Campbell & Powers, 2015). A second limitation is that we assessed female fitness only, and the effect on dispersal of pollen, an aspect of male fitness, is known to differ for some morphological traits in this species (Campbell, 1989).

In this study, selection based on seeds initiated per flower favored plants with increased emission of the dominant compound α‐pinene (β = 0.15 ± 0.04; compared with β = 0.17 ± 0.07 in Campbell *et al*., 2022a). In that previous study, levels of α‐pinene affected both the number of seeds initiated and seed predation, suggesting the selection arose from pollinators and seed predators.

A seed predator's oviposition strategy should favor plants that are likely to produce a large number of seeds. In *Ipomopsis*, total seed production increased and flies preferentially oviposited on plants with wide corollas and tall inflorescences (direct selection; Fig. 4a). Flies also laid more eggs on plants with higher nectar production and avoided those with high sabinene emissions. In the combined elastic net analysis, even more preferences for volatiles are evident (Fig. 4a). Egg‐laying preferences for wide corollas translated into higher fruit damage. This study adds to previous correlative and manipulative studies that found positive or neutral effects of floral display size or size of individual flowers on seed predation in *I. aggregata* (Brody, 1992; Brody & Mitchell, 1997) by suggesting that flies use olfactory as well as visual information.

The olfactory preferences of hummingbird pollinators for floral volatiles are just beginning to be explored (Kim *et al*., 2021). Whereas hummingbird‐pollinated flowers generally have little to no scent (unlike *Ipomopsis*; Knudsen *et al*., 2004), certain floral volatiles can attract or deter hummingbirds, at least when added to nectar (Kessler & Baldwin, 2006). In choice tests using artificial nectar scented with individual *Nicotiana* floral volatiles, hummingbirds (the late‐season *Ipomopsis* pollinator *Selasphorus rufus* and *Archilochus alexandri*) were repelled by three compounds also found in *I. aggregata* (limonene, methyl benzoate, and [(Z)‐hex‐3‐enyl] butanoate) and attracted by two *I. aggregata* sesquiterpenes, (*Z*)‐α‐bergamotene and β‐caryophyllene (comparing time nectaring relative to an unscented control; Kessler & Baldwin, 2006). Other *Ipomopsis* volatiles had no detectable effect: ocimene, hexan‐1‐ol, and (*Z*)‐3‐hexen‐1‐ol. The combined effect of both attractants and repellents may be neutral: The prior study calculated that these would balance out and another study found that whole floral odors added to feeders were not attractive to a different hummingbird (Núñez *et al*., 2021).

### Plasticity in floral traits has mixed effects on fitness

By integrating the data on plasticity with estimates of selection, we were able to test whether plasticity of floral traits is adaptive. A signature of adaptive plasticity for a floral volatile would be a change in volatile emission in a particular environment that also increased fitness in that environment. Overall, there were similar numbers of traits that showed maladaptive responses to the environmental shifts as adaptive responses (based on the sign of the change), and many traits were not under detectable selection, so overall fitness may not improve with floral trait plasticity to climate change.

### Conclusions

Floral volatile emissions were altered by two aspects of climate change, earlier snowmelt in the spring and changes in summer precipitation. These environmental factors also altered natural selection on individual volatiles, which could influence interactions with pollinators or seed predators. The responses of volatiles were complex, explainable by changes in soil moisture, and did not always correspond well between early snowmelt and reduced summer precipitation. This complexity highlights the need for more studies of how floral volatiles change with the environmental conditions plants will experience under future climate change.

## Competing interests

None declared.

## Author contributions

DRC proposed the initial idea for the study. HMB and DRC designed the study. All authors collected data. JMP analyzed the data. JMP and DRC drafted the manuscript.

## Supporting information


**Fig. S1** Timings of experimental treatments and measurements.
**Fig. S2** Number of floral volatile samples collected on each day.
**Fig. S3** Soil moisture throughout the experiment.
**Fig. S4** Correlations between floral traits.
**Fig. S5** Direct selection on traits in each treatment.
**Fig. S6** Effect of treatment on emissions of each compound class.
**Fig. S7** Relationship of plasticity to snowmelt timing vs plasticity to precipitation.
**Fig. S8** Effects of snowmelt date and precipitation on emissions of each compound class.
**Fig. S9** Effects of soil moisture on emissions of each compound class.
**Table S1** Number of plants sampled for each trait.
**Table S2** Emissions of each volatile compound.
**Table S3** Analyses of plasticity of emissions of each compound class.
**Table S4** Effects of treatments on natural selection for each compound class.
Please note: Wiley is not responsible for the content or functionality of any Supporting Information supplied by the authors. Any queries (other than missing material) should be directed to the *New Phytologist* Central Office.

## Data Availability

All data and code used to generate figures and tables are available at https://jmpowers.github.io/snow‐precip‐volatiles/.
