# Supplementary material for: Natural selection on floral volatiles and other traits can change with snowmelt timing and summer precipitation
Source: New Phytol. 2024 Sep 27;245(1):332–46. doi: 10.1111/nph.20157 (PMC11617657; doi:10.1111/nph.20157)

# New Phytologist Supporting Information

Article title: Natural selection on floral volatiles and other traits can change with snowmelt timing and summer precipitation

Authors: John M. Powers, Heather M. Briggs, Diane R. Campbell

Article acceptance date: 9 September 2024

## Table S1

Number of Ipomopsis aggregata plants sampled for volatiles, floral morphology, nectar, or all three, in each year and treatment combination.

| **Year** | **Precipitation** | **Snowmelt** | **Volatiles** | **Morphology** | **Nectar** | **All Traits** |
| --- | --- | --- | --- | --- | --- | --- |
| 2018 | Addition | Early | 21 | 20 | 17 | 16 |
| 2018 | Addition | Normal | 19 | 19 | 17 | 14 |
| 2018 | Control | Early | 21 | 32 | 19 | 9 |
| 2018 | Control | Normal | 26 | 37 | 19 | 12 |
| 2018 | Reduction | Early | 15 | 26 | 11 | 6 |
| 2018 | Reduction | Normal | 10 | 14 | 9 | 7 |
| 2019 | Addition | Early | 14 | 9 | 11 | 7 |
| 2019 | Addition | Normal | 37 | 35 | 36 | 28 |
| 2019 | Control | Early | 23 | 33 | 34 | 17 |
| 2019 | Control | Normal | 22 | 28 | 33 | 16 |
| 2019 | Reduction | Early | 16 | 14 | 14 | 11 |
| 2019 | Reduction | Normal | 19 | 23 | 23 | 17 |
| 2020 | Addition | Early | 22 | 21 | 19 | 13 |
| 2020 | Addition | Normal | 18 | 27 | 26 | 11 |
| 2020 | Control | Early | 31 | 38 | 34 | 16 |
| 2020 | Control | Normal | 30 | 30 | 27 | 12 |
| 2020 | Reduction | Early | 16 | 14 | 15 | 8 |
| 2020 | Reduction | Normal | 14 | 13 | 13 | 8 |
| **Total** | | | **374** | **433** | **377** | **228** |

##

## Table S2

Emissions of each volatile compound from *Ipomopsis aggregata* flowers (mean ± SD), with observed retention index (RI). Authentic standards were run for α-pinene, β-caryophyllene, and (Z)-3-hexen-1-ol. For statistical analyses, the C_11_ homoterpene (3E)-4,8-dimethylnona-1,3,7-triene is included with monoterpenes and the C_13_ carotenoid derivative pseudoionone is included with sesquiterpenes.

| **Class** | **RI** | **Compound** | **Emissions (ng/flower/hr)** |
| --- | --- | --- | --- |
| Aliphatics | 724 | 3-methylbutan-1-ol | 1.0 ± 1.8 |
|  | 851 | (Z)-hex-3-en-1-ol | 7.0 ± 12.2 |
|  | 865 | hexan-1-ol | 4.9 ± 5.0 |
|  | 956 | (E)-4-oxohex-2-enal | 1.2 ± 2.3 |
|  | 1003 | [(Z)-hex-3-enyl] acetate | 1.8 ± 3.6 |
|  | 1141 | unknown ester | 3.4 ± 7.5 |
|  | 1184 | [(Z)-hex-3-enyl] butanoate | 3.2 ± 8.0 |
| Benzenoids | 1096 | methyl benzoate | 0.6 ± 0.6 |
|  | 1141 | 2-methylbenzonitrile | 0.3 ± 0.2 |
| Monoterpenes | 933 | α-pinene | 42.2 ± 71.3 |
|  | 973 | sabinene | 5.9 ± 7.8 |
|  | 988 | β-myrcene | 4.8 ± 10.2 |
|  | 1010 | 3-carene | 10.1 ± 13.5 |
|  | 1029 | limonene | 16.7 ± 21.0 |
|  | 1035 | (Z)-β-ocimene | 15.6 ± 23.8 |
|  | 1047 | (E)-β-ocimene | 6.3 ± 12.1 |
|  | 1056 | γ-terpinene | 0.9 ± 1.5 |
|  | 1128 | (4E,6Z)-alloocimene | 0.2 ± 0.3 |
|  | 1195 | α-terpineol | 3.0 ± 4.6 |
|  | 1217 | verbenone | 3.2 ± 5.8 |
| Homoterpenes | 1115 | (3E)-4,8-dimethylnona-1,3,7-triene | 0.6 ± 1.3 |
| Sesquiterpenes | 1387 | α-copaene | 0.5 ± 1.3 |
|  | 1411 | petasitene | 1.8 ± 4.7 |
|  | 1424 | (Z)-α-bergamotene | 0.7 ± 0.9 |
|  | 1435 | β-caryophyllene | 4.1 ± 10.3 |
|  | 1454 | (E)-β-bergamotene | 1.0 ± 2.0 |
|  | 1470 | α-humulene | 0.6 ± 1.7 |
|  | 1605 | β-caryophyllene oxide | 1.0 ± 3.7 |
| Carotenoids | 1580 | pseudoionone | 0.5 ± 0.9 |

## Table S3

Analyses of plasticity of Ipomopsis aggregata emissions of each compound class. (A) Responses to treatments and year. (B) Responses to environmental variables of snowmelt date and estimated summer precipitation. (C) Responses to soil moisture at the plant level. (D) Responses to soil moisture at the subplot level. The table shows P-values derived from linear mixed models with random effects of plot and subplot (N = 346 plants for section C, otherwise N = 374).

|  | **Monoterpenes** | **Benzenoids** | **Sesquiterpenes** | **Aliphatics** |
| --- | --- | --- | --- | --- |
| **(A) Treatments** | **P** | | | |
| Year | **0.013** | **< 0.001** | **0.007** | 0.064 |
| Precip | 0.411 | 0.704 | 0.497 | 0.370 |
| Snow | 0.278 | 0.117 | 0.482 | **0.044** |
| Year*Precip | 0.278 | 0.326 | 0.120 | 0.421 |
| Year*Snow | 0.498 | 0.165 | 0.799 | 0.265 |
| Precip*Snow | 0.345 | 0.760 | 0.457 | 0.244 |
| Year*Precip*Snow | 0.704 | 0.221 | 0.784 | 0.476 |
| **(B) Environmental variables** | | | | |
| Snowmelt date | 0.668 | 0.196 | 0.655 | 0.362 |
| Summer precipitation | 0.566 | 0.286 | 0.301 | 0.662 |
| Snowmelt*Precipitation | 0.493 | 0.465 | 0.171 | 0.610 |
| **(C) Soil moisture measured at the plant level** | | | | |
| Year | **0.009** | **< 0.001** | **0.004** | **0.038** |
| Soil moisture | 0.657 | 0.116 | 0.444 | 0.733 |
| Year*Soil moisture | 0.106 | **0.020** | 0.130 | **0.042** |
| **(D) Soil moisture averaged across the season at the subplot level** | | | | |
| Year | **0.044** | 0.892 | 0.101 | 0.055 |
| Soil moisture | 0.543 | 0.903 | 0.367 | 0.247 |
| Year*Soil moisture | 0.052 | 0.764 | 0.083 | 0.092 |

##

## Table S4

Effects of snowmelt and precipitation treatments and the year on natural selection for Ipomopsis aggregata floral emission rates of each compound class. Values listed are P-values derived from linear models for each of the four fitness measures that included all of the terms shown. Effects of the years and treatments indicate direct effects on fitness, effects of each compound class indicate overall selection on emissions of that class, and interactions between the year or treatments and a compound class indicate that selection varies by year or treatment.

|  | **Fly eggs per flower** | **Prop. uneaten fruits** | **Total seeds** | **Seeds initiated per flower** |
| --- | --- | --- | --- | --- |
|  | P | | | |
| Year | **0.000** | **0.015** | 0.120 | **0.000** |
| Precip | 0.718 | 0.239 | 0.588 | 0.156 |
| Snowmelt | 0.455 | 0.897 | 0.122 | **0.050** |
| Monoterpenes | 0.170 | 0.651 | 0.054 | 0.314 |
| Sesquiterpenes | 0.609 | 0.088 | 0.320 | 0.910 |
| Benzenoids | 0.216 | 0.106 | **0.021** | 0.196 |
| Aliphatics | 0.523 | 0.327 | 0.789 | 0.775 |
| Year*Precip | 0.993 | 0.362 | **0.036** | **0.004** |
| Year*Snowmelt | 0.565 | 0.960 | **0.002** | 0.217 |
| Precip*Snowmelt | 0.669 | 0.416 | 0.484 | 0.369 |
| Snowmelt*Monoterpenes | 0.581 | 0.442 | 0.135 | 0.420 |
| Precip*Monoterpenes | 0.458 | 0.387 | 0.790 | 0.290 |
| Year*Monoterpenes | 0.228 | **0.012** | 0.442 | 0.834 |
| Snowmelt*Sesquiterpenes | 0.124 | 0.052 | 0.496 | **0.048** |
| Precip*Sesquiterpenes | 0.720 | **0.001** | 0.594 | 0.345 |
| Year*Sesquiterpenes | 0.375 | 0.060 | 0.779 | 0.998 |
| Snowmelt*Benzenoids | 0.541 | 0.782 | 0.587 | 0.982 |
| Precip*Benzenoids | 0.686 | 0.124 | 0.619 | 0.352 |
| Year*Benzenoids | 0.391 | 0.484 | 0.210 | 0.328 |
| Snowmelt*Aliphatics | 0.365 | 0.228 | **0.048** | 0.132 |
| Precip*Aliphatics | 0.071 | 0.627 | 0.794 | 0.226 |
| Year*Aliphatics | 0.398 | 0.564 | 0.609 | 0.974 |
| Year*Precip*Snowmelt | 0.089 | 0.227 | 0.618 | 0.807 |

##

## Figure S1

Timings of experimental treatments and measurements across the three years of the study. An avalanche disrupted snow cloth placement in 2019. The mean snowmelt timing is given for the early snowmelt plots (first point) and the unmanipulated snowmelt plots (second point). The soil moisture dates are for measurements at the subplot scale, but plant-level measurements were taken during volatiles sampling.


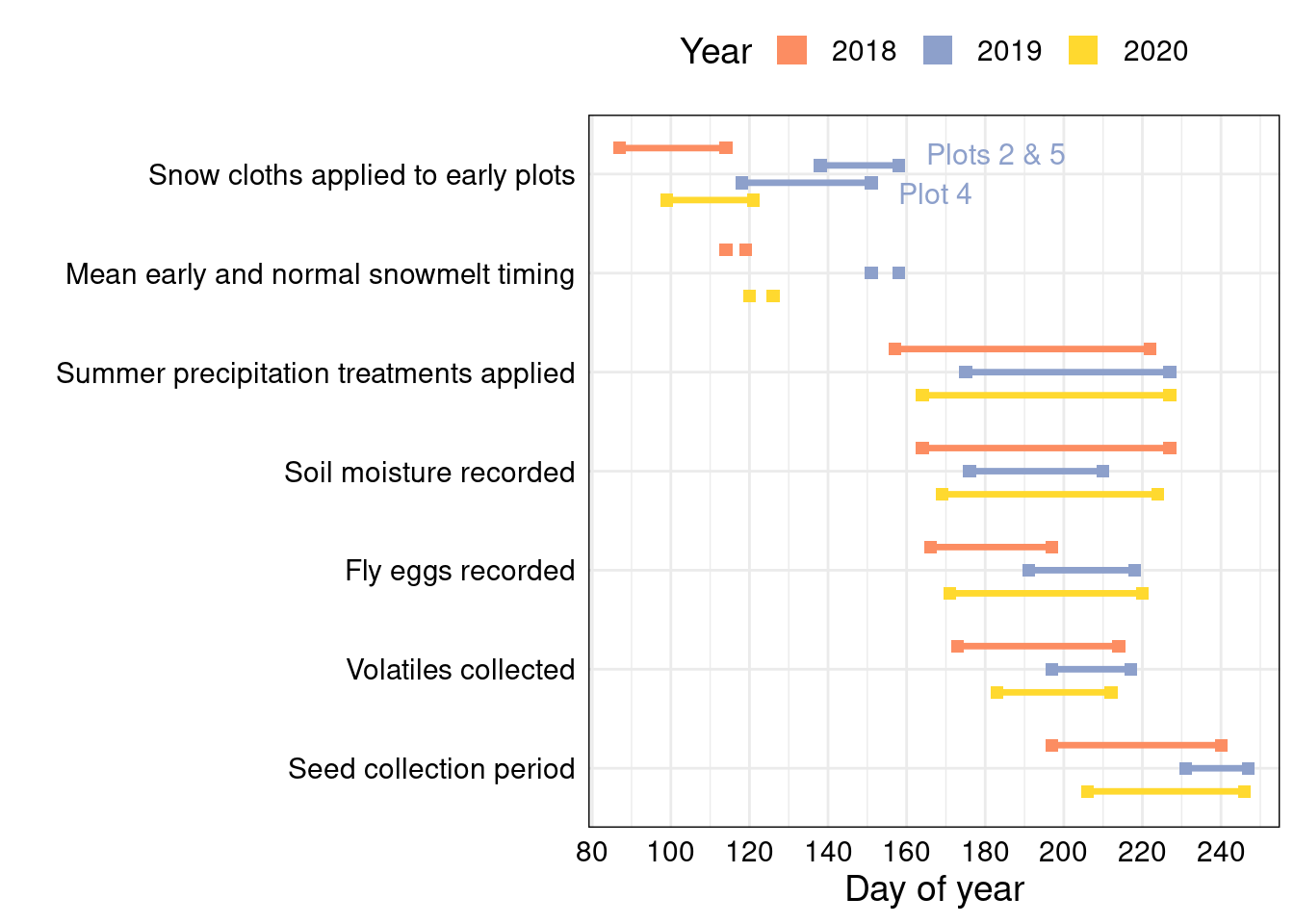


##

## Figure S2

Number of Ipomopsis aggregata floral volatile samples collected from each treatment (indicated by color) on each day of sampling.


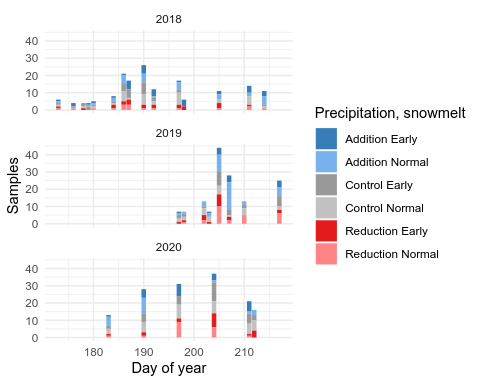


##

## Figure S3

Points show soil moisture (volumetric water content, %VWC) measured next to each Ipomopsis aggregata flowering plant on the day it was sampled for volatiles, colored by precipitation treatment. Thick lines show the average soil moisture next to each plant. Thin lines show the average soil moisture measured at the corners and center of each subplot. Green bars show daily precipitation at a nearby weather station (see Powers et al. 2021).


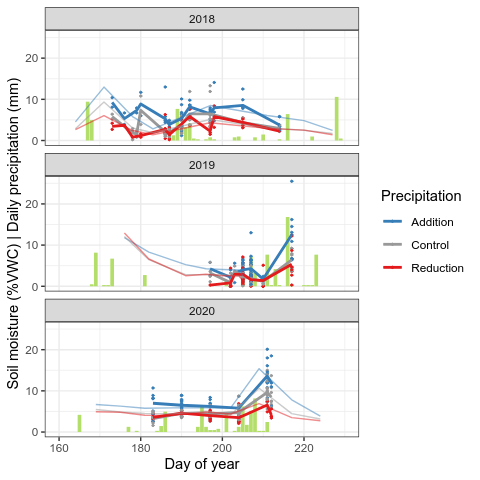


##

## Figure S4

Pearson correlations between Ipomopsis aggregata floral traits, including emission rates of floral volatiles. Positive correlations are indicated in red and negative correlations in blue. Traits are clustered by the similarity of their correlations. For volatiles, the compound class is indicated at the margins.


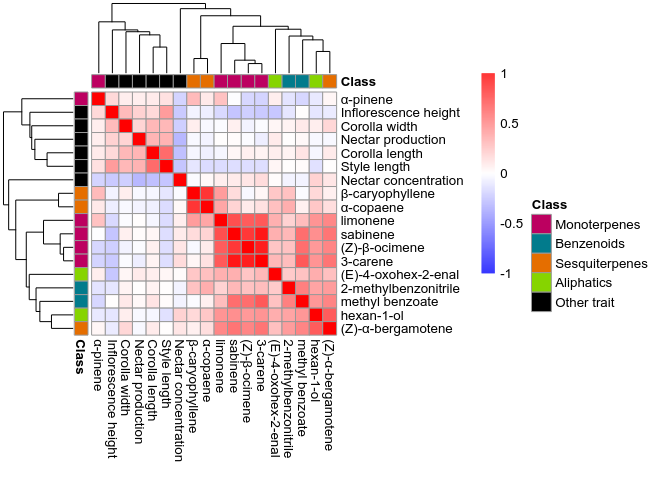


##

## Figure S5

Direct selection on Ipomopsis aggregata traits, including floral volatile emissions, in (A) each snowmelt treatment, and (B) each precipitation treatment. Estimates are based on the subset of plants for which all traits were measured, in an elastic net regression run either separately on volatiles and other traits (circles) or a combined analysis of all traits (diamonds).

A.


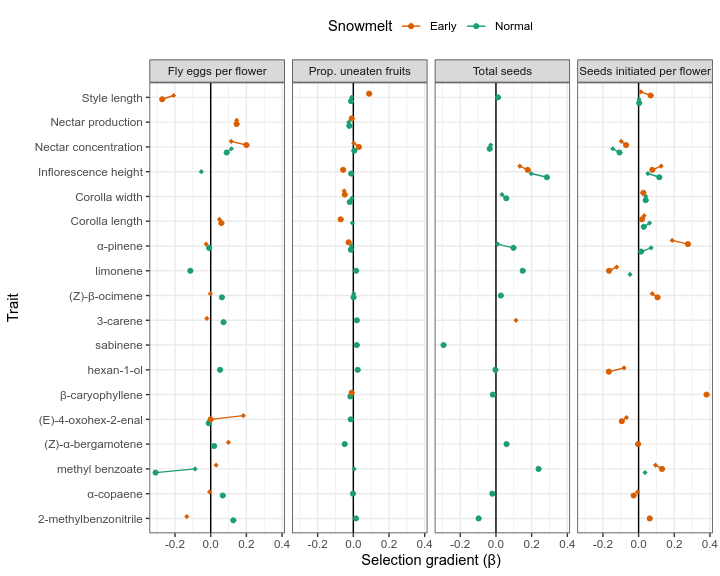


B.


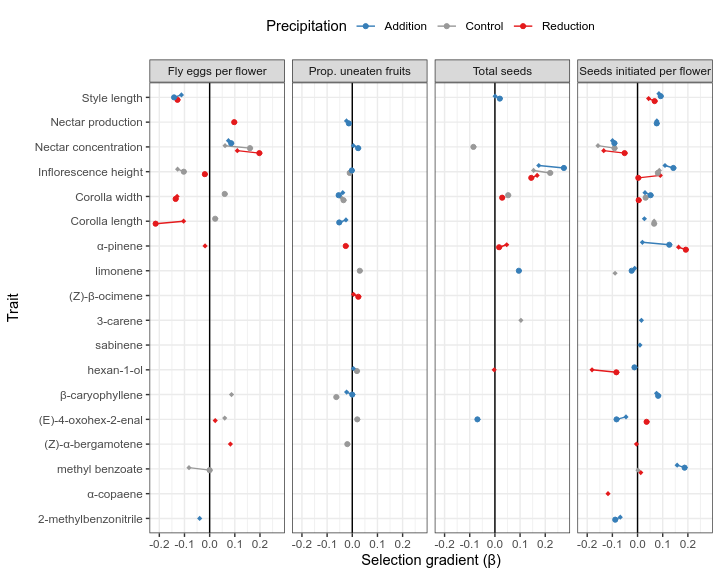


##

## Figure S6

Ipomopsis aggregata floral volatile emissions divided by class of chemical compound in each combination of precipitation treatment, snowmelt treatment, and year. Boxplots show the median and first and third quartiles, with outliers as points. The emissions axis is square-root spaced.


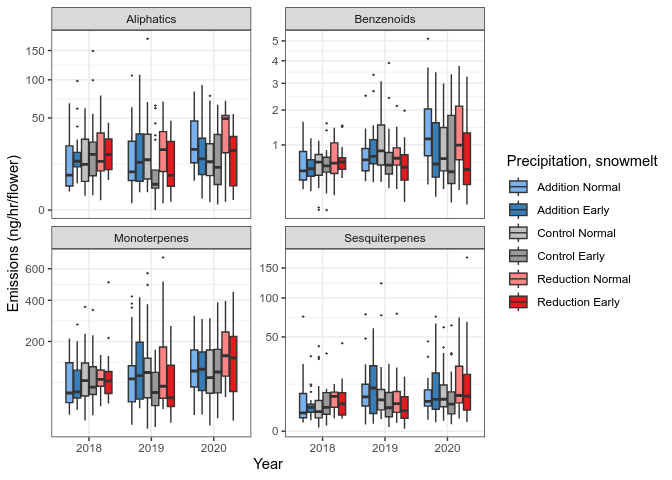


##

## Figure S7

The relationship of plasticity of Ipomopsis aggregata floral traits to snowmelt timing versus plasticity to precipitation. Plasticity is calculated as the fold change in the trait under one treatment (early snowmelt or reduced precipitation) relative to another treatment (normal snowmelt or additional precipitation). Error bars represent standard errors. Points for floral volatiles are sized by their mean emissions.


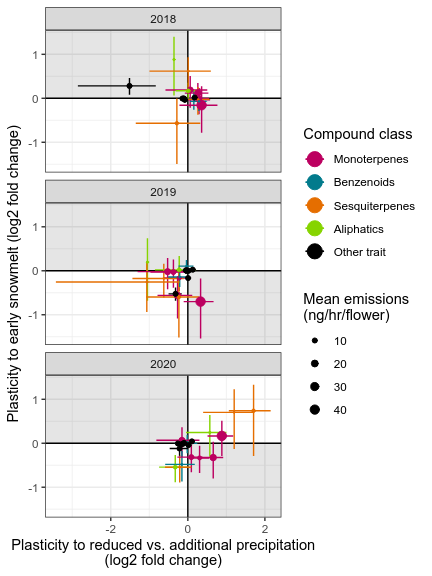


##

## Figure S8

Model results for the effects of the snowmelt date and estimated summer precipitation on Ipomopsis aggregata floral volatile emission rates. Lines of each color show the estimated marginal trends for the amounts of summer precipitation listed in the key. Points show the emissions of each plant, colored by the estimated summer precipitation.


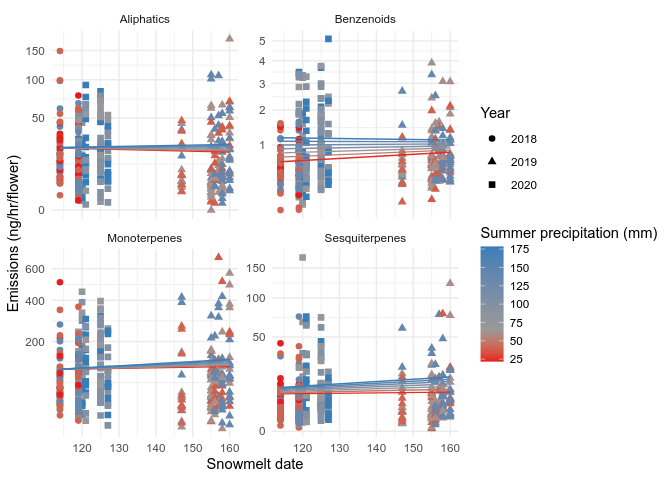


##

## Figure S9

The effects of soil moisture (volumetric water content, VWC) on Ipomopsis aggregata floral emissions of each compound class. Soil moisture was measured at five positions in each subplot, averaged across the season. Curves show loess fits in each year.


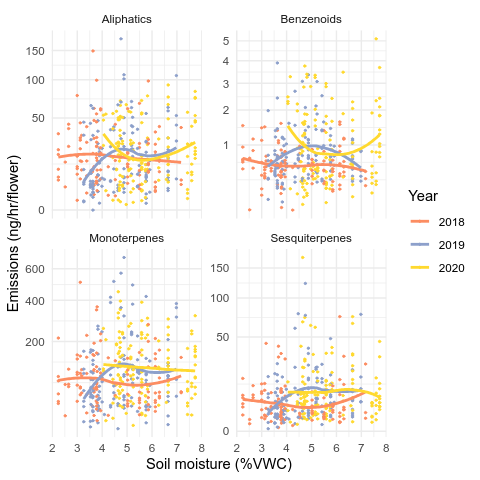

Supplement: Supplementary file 1 — Fig. S1 Timings of experimental treatments and measurements. Fig. S2 Number of floral volatile samples collected on each day. Fig. S3 Soil moisture throughout the experiment. Fig. S4 Correlations between floral traits. Fig. S5 Direct selection on traits in each treatment. Fig. S6 Effect of treatment on emissions of each compound class. Fig. S7 Relationship of plasticity to snowmelt timing vs plasticity to precipitation. Fig. S8 Effects of snowmelt date and precipitation on emissions of each compound class. Fig. S9 Effects of soil moisture on emissions of each compound class. Table S1 Number of plants sampled for each trait. Table S2 Emissions of each volatile compound. Table S3 Analyses of plasticity of emissions of each compound class. Table S4 Effects of treatments on natural selection for each compound class. Please note: Wiley is not responsible for the content or functionality of any Supporting Information supplied by the authors. Any queries (other than missing material) should be directed to the New Phytologist Central Office. [file NPH-245-332-s001.docx]
